# Assessment of oral health status in a population of Moroccan children with type 1 diabetes

**DOI:** 10.3389/froh.2025.1638222

**Published:** 2025-09-12

**Authors:** Fatima Ezzahra Zidane, Soukaina Rouijel, Rachid Fawzi

**Affiliations:** 1 International Faculty of Dental Medicine, Health Sciences Research Center (CReSS), College of Health Sciences, International University of Rabat, Sala Al Jadida, Morocco; 2 Private University of Marrakech-Marrakesh Private Hospital, Marrakech, Morocco

**Keywords:** diabetes, gingival index, plaque index, gingivitis, calculus deposit

## Abstract

**Background:**

Type 1 diabetes has become a major health problem in Morocco, given its incidence and high prevalence. The repercussions linked to this disease are various particularly on oral health. The aim of this study is to assess the impact of type 1 diabetes on the oral health of Moroccan children and to evaluate the influence of oral health on blood sugar control.

**Methods:**

In this cross-sectional study 100 children and adolescents with type 1 diabetes aged 3–17 years took part. The non-diabetic group was composed of children and adolescents in overall good health, enrolled in public schools and part of the national dental prevention program, selected to match the diabetic group in terms of number, age and age group. The variables collected were socio-demographic, related to diabetes and related to oral health. The clinical examination revealed variables related to the oral state (DMFT/dmft index, plaque index, gingival index). The Chi-square test was used to compare categorical variables, *t*-test to compare quantitative variables and Anova test to compare between three groups. We performed linear regression to study the factors associated with dental caries, plaque index and gingival index.

**Results:**

The average age was 9.61, 2.65 of whom 48.5% were male, aged from 3 to 17 years. All participants had at least one dental cavity but the average DMFT/dmft index was higher in diabetic subjects (6.13 ± 3.26). Patients with type 1 diabetes showed a decline in the majority of the oral health metrics examined. Statistically significant differences were found between diabetic and non-diabetic groups in terms of calculus deposition and degree of inflammation (with respectively *p* = 0.001, *p* = 0.022). A significant difference was observed for gingival inflammation which was more pronounced in children with uncontrolled type 1 diabetes (*p* = 0,043). In linear regression a relation between the plaque index and tooth brushing in univariate analysis *p* < 0.001.

**Conclusion:**

The results of our study indicated that patients with type 1 diabetes experienced a decline in most of the oral health metrics assessed. This may suggest that both diabetes and its control have a significant impact on oral health.

## 1 Introduction

Diabetes is a chronic metabolic disorder characterized by prolonged hyperglycemia, which results from either an absolute or relative deficiency of insulin. This insulin deficiency can be attributed to a combination of genetic and environmental factors (1, 2). Hyperglycemia, or elevated blood glucose levels, is a hallmark of poorly controlled diabetes and, over time, can cause substantial damage to multiple organ systems, particularly the nervous and cardiovascular systems (3). These complications are diverse and affect many areas of the body, including the oral cavity. Chronic hyperglycemia can lead to a range of oral health issues, such as xerostomia, which impairs the antimicrobial action of saliva. Additionally, individuals with type 2 diabetes are at a higher risk for periodontal disease, mucosal infections, sensory disturbances and even tooth loss, all of which significantly impact their quality of life (3, 4).

Over the past decades, many countries have observed a rise in both the incidence and the overall prevalence of the disease (2). In Morocco, according to the Minister of Health, between 2011 and 2023, the number of diabetics increased from 1.5 million individuals to more than 2 million. This rise in diabetes cases comes amid Morocco's changing demographics, with a population that has grown from approximately 32 million people in 2011 to an estimated 37 million in 2023 (5).

Globally, Type 1 diabetes represents approximately 5%–10% of all diabetes cases. This proportion is likely similar in Morocco, implying that between 100,000 and 200,000 people in Morocco may be affected by the condition (5). The prevalence of diabetes may have approached 15%, with a rising incidence of related complications such as cardiovascular diseases, kidney problems, and blindness positioning diabetes as a significant public health concern in Morocco (5).

Given the rising prevalence of type 1 diabetes in children and adolescents, its complications and its chronic nature, it is crucial to assess the broader health impacts, especially concerning oral health.

If complications of type 2 diabetes on oral health are widely described in the literature, what can we say about the effect of type 1 diabetes on oral health and the impact of oral health on blood sugar control, it seems that we are talking more and more about the existence of a bidirectional association.

This study aims to evaluate the impact of type 1 diabetes on the oral health of Moroccan children and to assess the effect of oral health on blood sugar control.

## 2 Materials and methods

### 2.1 Study population

This cross-sectional study included 100 children with type 1 diabetes attending the Young Diabetic House in Rabat, along with 100 healthy children enrolled in public schools and participating in the national dental prevention program.

The sample was a convenience sample, as the study was conducted over a six-month period from December to June. During this time, we recruited 100 diabetic and 100 non-diabetic children.

Inclusion criteria:

- Children aged 3–17 years.
- Children and adolescents whose parents provided written informed consent to participate in the study.
- For the diabetic group, only patients with a confirmed diagnosis of type 1 diabetes were included.
- For the non-diabetic group, children in good general health without conditions affecting oral health were included.

The study evaluated diabetes control, dental caries, gingival health, and oral hygiene status using the following measures: glycated hemoglobin (HbA1c) for diabetes control, the DMFT/dmft index for dental caries, the Gingival Index (GI) for gingival health, and the plaque index (PI) to assess oral hygiene.

### 2.2 Conduct of the investigation

Following approval from the dean of our university and the Institutional Review Board (CUMD/FIMD 003/20/24/Approval/20/24), the study was initiated. An oral explanation of the objectives of the study was provided to both the children and their parents to obtain their written informed consent. Upon receipt of consent, the clinical examination was conducted.

The informed consent form invited parents and children to participate in a study investigating the impact of type 1 diabetes on oral health. Participation was entirely voluntary, with all data collected anonymously and treated with strict confidentiality. The study posed no direct risks to participants, who retained the right to withdraw at any point without consequence.

### 2.3 Collection of data and variables studied

For our study, two forms were developed:

- One form for diabetic patients (Supplementary Appendix 1)
- A second form for “ non-diabetics,” similar to the first, but excluding the section related to diabetes (Supplementary Appendix 2).

Each form was composed of two sections:

The questionnaire:

This section was designed to gather various types of variables:

- Sociodemographic, educational, and economic variables:
  - Age
  - Gender
  - Place of birth
  - Child's level of education: preschool, primary school, secondary school
  - Father's level of education: none, primary, secondary, or university
  - Mother's level of education: none, primary, secondary, or university
- General health variables:
  - Medical history
  - Surgical history
  - Allergies
  - Age at diagnosis of diabetes
  - Duration of diabetes: time elapsed since diagnosis
  - Number of insulin injections per day
  - Average glycated hemoglobin (HbA1c) level:
    ▪ <7.5% (controlled diabetes)
    ▪ Between 7.5% and 9.5% (uncontrolled diabetes)
    ▪ 9.5% (uncontrolled diabetes)
  - Controlled or uncontrolled diabetes
- Oral hygiene variables:
  - Age at which tooth brushing began
  - Frequency of toothbrushing (once a day, twice a day, three times a day, etc.)
  - Duration of brushing
- Oral health variables:
  - History of dental care
  - Age of child at first dental consultation
  - Parents’ knowledge regarding the relationship between oral health and diabetes
  - Date of last dental consultation
  - Reasons for dental consultation
  - Type of dental care received
  - Reasons for not seeking dental care
- Eating habits variables:
  - Balanced diet: variety of foods (proteins, vitamins, etc.)
  - Snacking habits: frequency and types of snacks (if applicable)
  - For the diabetic group, questions regarding eating habits before and after the diagnosis of diabetes

Clinical examination:

The oral health examination was conducted for each subject in both groups to collect data on their oral health status. This included:

- Periodontal status variables:
  ▪ LOE and Silness plaque index (6)
  ▪ Gingival index (6)
- Dental condition variables:
  ▪ DMFT/dmft index (decayed, missing, or filled teeth) permanent/primary
- Other oral health variables:
  ▪ Presence of calculus
  ▪ Condition of the mucosa
  ▪ Molar Incisor Hypomineralization (MIH) (7)

### 2.4 Data processing

Statistical analysis was performed using SPSS version 20.0.0 (Statistical Package for the Social Sciences).

Quantitative variables were expressed as means and standard deviations, while categorical variables were expressed as frequencies and percentages.

To analyze associations between different variables, the following statistical tests were used:

- The chi-square test for comparing categorical variables.
- The student's *t*-test and analysis of variance (ANOVA) for comparing quantitative variables between two and multiple independent groups, respectively.
- Linear regression to study the factors associated with dental caries, plaque index and gingival index.
- A significant level of 5% was set for all statistical tests.

## 3 Results

Following the defined inclusion criteria, the non-diabetic group comprised children and adolescents in good general health with no treatments that could influence oral health.

For a more detailed statistical analysis, which would help assess the risk and carious context, as well as evaluate oral health status, we divided both populations into three age groups:

- 3–5 years
- Over 5 years to 12 years
- Over 12 years

These groups corresponded to the stages of primary dentition, mixed dentition, and permanent dentition, respectively.

### 3.1 Descriptive results

#### 3.1.1 Description of the population according to socio-demographic characteristics

The diabetic group comprised 53% boys and 47% girls, aged 3–17 years, with a mean age of (9.83 ± 2.73). The age group of 6–12 years was the most prevalent in both diabetic children and adolescents, as well as in the non-diabetic group, accounting for 76% of participants (Table 1).

**Table 1:** Distribution of participants according to socio-demographic characteristics.

Variable	Diabetic group (*n* = 100)	Non-diabetic group (*n* = 100)
Age^a^	9.83 ± 2.73	9.39 ± 2.57
Gender^b^		
Female	47 (47)	56 (56)
Male	53 (53)	44 (44)
Age groups^b^		
3–5 years	2 (2)	2 (2)
Over 5–12 years	76 (76)	76 (76)
Over 12 years	22 (22)	22(22)

a Expressed as “mean ± SD”.

b Expressed as “*n* (%)”.

#### 3.1.2 Description of the population according to age of discovery of diabetes

58% of diabetic subjects reported having discovered diabetes before age of 10.

#### 3.1.3 Description of the population according to glycemic control

22% of diabetic subjects had a glycated hemoglobin level below 7.5%, of which all were classified as having controlled diabetes.

### 3.2 Analytic results

#### 3.2.1 Comparison of oral health between diabetic and non-diabetic groups

In this study, all participants had at least one dental cavity. The average DMFT/dmft index for diabetic subjects was (6.1 ± 3.26). While for non-diabetics (5.85 ± 3.58). Both reflect a high level of dental caries.

**Figure 1:**
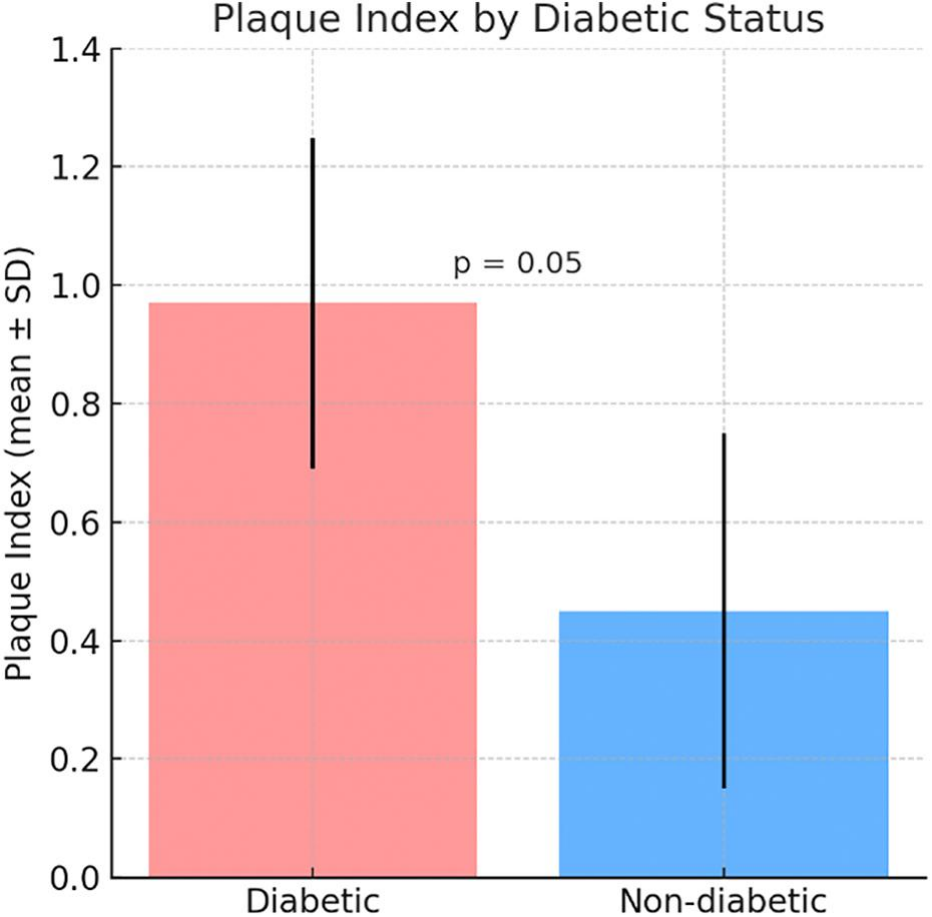
Comparative mean plaque Index between diabetic and non-diabetic participants. Bars represent mean ± standard deviation (SD).

**Figure 2:**
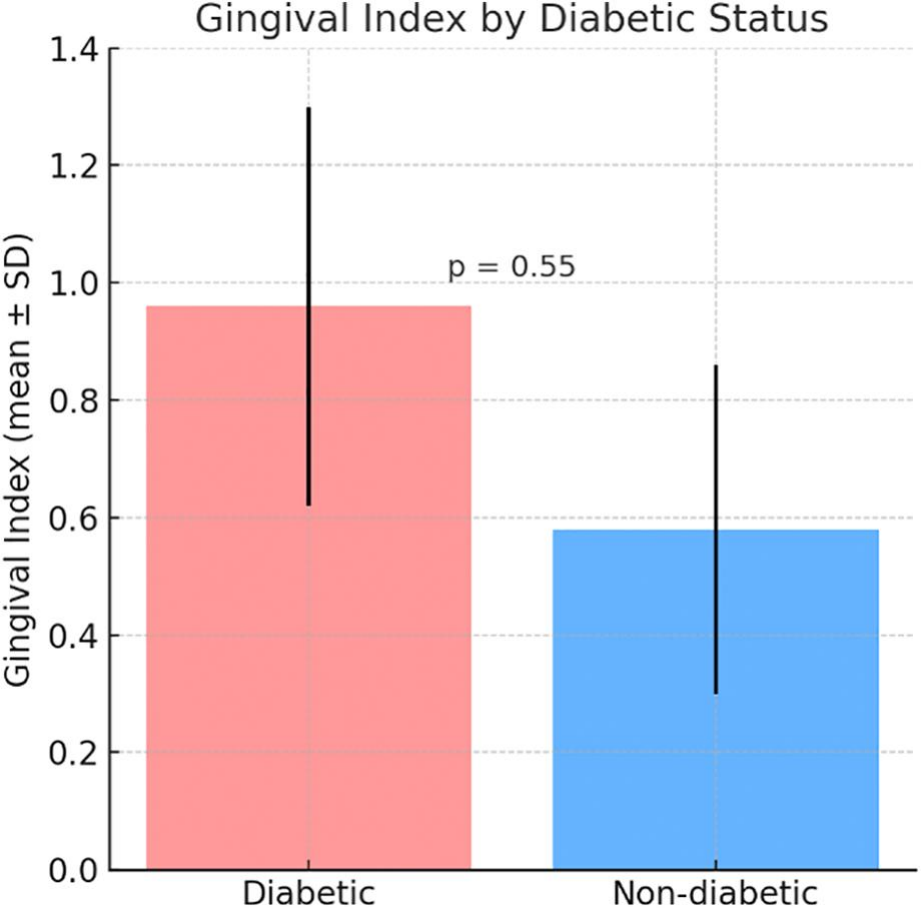
Comparative mean gingival Index between diabetic and non-diabetic participants. Bars represent mean ± standard deviation (SD).

The Plaque Index was higher in the diabetic group (0.97 ± 0.28) compared to the non-diabetic group (0.45 ± 0.30) (Figure 1). The Gingival Index was also higher in the diabetic group (0.96 ± 0.34) compared to the non-diabetic group (0.58 ± 0.28) (Figure 2) but the differences were not statistically significant, with respectively (*p* = 0.05, *p* = 0.55) (Figures 1,2).

84% of both groups exhibited gingival inflammation. Calculus deposits were absent in 60% of diabetic subjects and 82% of non-diabetics.

Statistically significant differences were identified between diabetic and non-diabetic groups in terms of calculus deposition and degree of inflammation, with respectively *p* = 0.001 and *p* = 0.022 (Table 2).

**Table 2:** Comparison of oral health between diabetic and non-diabetic groups.

Variables	Diabetes		*p*
	Diabetic group (*n* = 100)	Nondiabetic group (*n* = 100)	
DMFT	6.1 ± 3.26	5.85 ± 3.58	0.6
Plaque index +	0.97 ± 0.28	0.45 ± 0.30	0.05
Gingival index +	0.96 ± 0.34	0.58 ± 0.28	0.55
Calculus deposit^a^			0.001
No calculus	60 (42.3)	82 (57.7)	
Mild deposit	38 (67.9)	18 (32.1)	
Very abundant	2 (100)	0 (0)	
Degree of inflammation^a^			0.022
Mild inflammation	45 (53.5)	59 (70.23)	
Moderate inflammation	37 (44.04)	25 (29.76)	
Severe inflammation	2 (2.38)	0(0)	

Kh2 and t of student + Expressed as “mean ± SD”.

a Expressed as “*n* (%)”.

Additionally, a correlation was observed between blood sugar levels (HbA1c) and calculus deposit, as well as between HbA1c and the severity of inflammation respectively *p* < 0.001, *p* = 0.01 (Table 3).

**Table 3:** Correlation in diabetic group.

Variables	Correlation coefficient (r)	*P*
HbA1c/DMFT	−0.36	−0.36
HbA1c/plaque index	1.13	0.05
HbA1c/gingival index	0.42	0.55
HbA1c/tartar deposit	−0.25	<0.001
HbA1c/gingival inflammation	−0.19	0.01

#### 3.2.2 Comparison of oral health between controlled and uncontrolled diabetic groups

No statistically significant differences were observed between the two groups in terms of the oral health variables, except for gingival inflammation which was significantly more pronounced in children with uncontrolled type 1 diabetes and statistically significant (*p* = 0,043) (Table 4).

**Table 4:** Comparison of oral health between controlled and uncontrolled diabetic groups.

Variables	Controlled diabetes		*P*
	Yes	No	
DMFT +			0.59
	5.77 ± 3.37	6.19 ± 3.24	
Plaque index	0.96 ± 0.27	0.97 ± 0.28	0.93
Gingival Index +	0.92 ± 0.38	0.97 ± 0.33	0.56
Gingival inflammation^a^			0.043
Yes	15 (68.2)	69 (88.5)	
No	7 (31.8)	9 (11.5)	
Calculus deposit^a^			0.42
No calculus	11 (50)	49 (62.8)	
Mild deposit	11 (50)	27 (34.6)	
Very abundant	0 (0)	2(2.6)	

Kh2 and t of student + Expressed as “mean ± SD”.

a Expressed as “*n* (%)”.

There are no statistically significant differences observed between the three groups regarding gingival inflammation (Table 5)

**Table 5:** Effects of diabetes on gingival inflammation.

Variable	Controlled diabetes		Nondiabetic	*p*
	Yes	No		
Gingival index +	0.92 ± 0.38	0.96 ± 0.33	0.98 ± 0.28	0.06

Anova + Expressed as “mean ± SD”.

In the linear regression analysis investigating the associations between DMFT index, plaque index and gingival index, along with controlled diabetes, gender, age and tooth brushing, a statistically significant relationship was observed between plaque index and tooth brushing in the univariate analysis *p* < 0.001 (Table 7). However, no other statistically significant associations were found (Tables 6–8).

**Table 6:** Factors associated with dental caries in diabetic group .

Variables	DMFT index					
	Univariate analysis			Multivariate analysis		
	β	CI 95%	*p*	β	CI 95%	*p*
Controlled diabetes						
Yes- no	0.022	[−0.02, 0.06]	0.31	0.022	[−0.02, 0.068]	0.34
Gender						
F-M	−0.02	[−0.06, 0.008]	0.13	−0.03	[−0.07, 0.003]	0.07
Age groups						
6–12–3–5	−0.02	[−0.15, 0.10]	0.70	−0.01	[−0.14, 0.12]	0.84
Up to 12–3–5	−0.06	[−0.19, 0.07]	0.35			
	−0.05	[−0.19, 0.09]	0.48			
Teeth brushing						
Yes- no	0.01	[−0.03, 0.07]	0.48	0.01	[−0.03, 0.068]	0.45

**Table 7:** Factors associated with plaque index in diabetic group .

Variables	Plaque index					
	Univariate analysis			Multivariate analysis		
	β	CI 95%	*p*	β	CI 95%	*p*
Controlled diabetes						
Yes- no	−0.044	[−0.14, 0.13]	0.94	−0.008	[−0.14, 0.12]	0.90
Gender						
F-M	0.02	[−0.08, 0.14]	0.36	−0.23	[−0.37, 0.08]	0.002
Age groups						
6 to 12y—3 to 5 y	−0.24	[−0.64, 0.14]	0.21	−0.18	[−0.57, 0.21]	0.36
Up to 12y—3 to 5 y	−0.11	[−0.5, 0.29]	0.58	−0.05	[−0.45, 0.35]	0.79
Teeth brushing						
Yes- no	−0.24	[−0.38, −0.10]	<0.001	0.23	[−0.38, −0.08]	0.002

**Table 8:** Factors associated with gingival index in diabetic group.

Variables	Gingival Index					
	Univariate analysis			Multivariate analysis		
	β	CI 95%	*p*	β	CI 95%	*p*
Controlled diabetes						
Yes- no	−0.041	[−0.2, 0.12]	0.61	−0.04	[−0.21, 0.12]	0.59
Gender						
F-M	0.03	[−0.10, 0.17]	0.60	0.066	[−0.07, 0.20]	0.34
Age groups						
6–12 years—3–5 years	−0.05	[−0.54, 0.44]	0.82	0.002	[−0.49, 0.5]	0.99
Up to 12 years—3–5 years	0.03	[−0.46, 0.54]	0.88	0.07	[−0.44, 0.58]	0.78
Teeth brushing						
Yes- no	−0.27	[−0.45, −0.09]	0.003	0-0.27	[−0.46, −0.08]	0.004

## 4 Discussion

### 4.1 Diabetes and dental caries

The comparison between the two groups in this study revealed that the mean DMFT/dmft index of diabetic children was slightly higher than that of the non-diabetics with respectively (6.1 ± 3.26), (5.85 ± 3.58) though the difference was statistically insignificant (*p* = 0.6).

The results of this study align with some existing literature, but there is notable variation in the findings of other research. Some studies have reported that the mean DMFT/dmft index in diabetic group is higher than that in non-diabetic group, with statistically significant differences (*p* < 0.001) (8, 9). These studies suggest that diabetes may contribute to an increased risk of dental caries, potentially due to factors such as higher glucose levels in saliva, which may promote bacterial growth and impaired oral hygiene practices among diabetic children.

In contrast, other studies have found no significant difference between the mean DMFT index of diabetic and non-diabetic groups (10).

A meta-analysis published in 2020 (11) reached conclusions like ours; with type 1 diabetic patients showing a higher mean DMFT index compared to non-diabetic individuals. However, like our study, this meta-analysis found that the difference was statistically insignificant. The variations in these findings may be attributed to differences in study design, sample sizes, geographic locations and the methods used to assess oral health.

Quantitative and qualitative changes in saliva are commonly observed in individuals with uncontrolled diabetes (12). Such alterations can be attributed to the prolonged exposure of diabetic patients to changes in salivary composition, including an increase in glucose and calcium levels, as well as a decrease in the pH of saliva. These factors create a favorable environment for the proliferation of cariogenic bacteria and contribute to an increased risk of dental caries and oral infections in diabetic individuals (13).

The primary goal of both preventive and curative treatments for diabetes is to achieve optimal glycemic control, which is crucial in preventing long-term degenerative complications and ensuring a good quality of life. Maintaining controlled blood sugar levels is essential for minimizing the risk of complications (14).

In our study, only 22% of the children and adolescents had HbA1c below 7.5%, which is considered an indicator of controlled diabetes. This is surprising given that insulin is provided free of charge at health centers and most of the patients are being followed regularly. The paradox could be attributed to several factors, including patient fatigue in managing the disease or inconsistency in adherence to treatment regimens.

Our findings also indicated that subjects with a high DMFT/dmft index tended to have uncontrolled diabetes, although the difference was not statistically significant (*p* = 0.59).

In a study conducted in Japan (15), children who developed dental caries had the highest HbA1c level and therefore uncontrolled diabetes with a statistically insignificant difference as well. A meta-analysis (11) also found results similar to ours. No significant difference was found between type 1 controlled and uncontrolled diabetics.

In the linear regression analysis investigating the associations between DMFT index and controlled diabetes, along with gender, age, and tooth brushing, no statistically significant relationship was observed (16).

### 4.2 Diabetes and periodontium

In the literature, the relationship between diabetes and periodontal disease has often been discussed. Indeed, in the presence of uncontrolled diabetes, the risk of developing gingivitis or periodontal disease is real by promoting changes in the periodontal sphere. Thus, in children whose diabetes are uncontrolled, we often observe gums redder than normal, more swollen, sometimes hypertrophic and painful (13). The modification of oral microbial flora promotes oral infections, especially gingivitis.

In our examined population, all the index of periodontal disease were influenced by diabetes:

Figure 1 shows that the Plaque Index was higher in the diabetic group (0.97 ± 0.28) compared to the non-diabetic group (0.45 ± 0.30), indicating greater plaque accumulation in diabetic patients. Similarly, Figure 2 illustrates a higher Gingival Index in the diabetic group (0.96 ± 0.34) vs. the non-diabetic group (0.58 ± 0.28). However, these differences were not statistically significant, with *p*-values of 0.05 for the Plaque Index and 0.55 for the Gingival Index.

Our study also found a high prevalence of inflammatory gum tissue of 84% as well in diabetic subjects as in the non-diabetic group (*p* = 0.022). Calculus deposit where diabetic subjects presented more calculus than non-diabetic subjects (*p* = 0.001).

Same results found in a study on the prevalence and incidence of periodontal disease in diabetics subjects (17). Despite plaque control and frequent tooth brushing, gingivitis was found significantly more severe in type 1 diabetes compared to non-diabetic group (18).

There are several research studies that support the hypothesis of periodontitis that occurs more frequently in diabetics with uncontrolled blood sugar (19, 20).

Our results show the most unfavorable periodontal index concerning uncontrolled diabetes. A higher gingival index, plaque index and calculus deposit among uncontrolled diabetic children compared to controlled diabetic children but with statistically insignificant differences *p* = 0.56, *p* = 0,93, *p* = 0,42. Additionally, a correlation was observed between blood sugar levels (HbA1c) and calculus deposit, as well as between HbA1c and the severity of inflammation respectively *p* < 0.001, *p* = 0.01.

Prevalence of gingivitis was observed in the two groups, with a statistically significant association *p* = 0.043. Regarding the effects of diabetes on gingival inflammation, no statistically significant differences were observed between the three groups: controlled, uncontrolled and non-diabetics *p* = 0.06. Regarding the associations of plaque index and gingival index with controlled diabetes, gender, age and tooth brushing; it appears that there is a statistically signification relation between the plaque index and tooth brushing in univariate analysis *p* < 0.001, beside this no statistically significant association was observed.

A meta-analysis published in the journal clinic periodontal (21), had concluded that periodontitis has a significant impact on diabetes control, incidence and complications. Available data on epidemiological evidence has established that periodontitis has a significant effect on the problems associated with type 2 diabetes.

But what about type 1 diabetes, do people with periodontitis and type 1 diabetes have poorer glycemic control than those with diabetes and better periodontal health?

The consensus report and guidelines from the joint workshop on periodontal diseases and diabetes, developed by the International Diabetes Federation and the European Federation on Periodontology, highlighted the strong relationship between periodontitis and diabetes (22). They concluded that poor glycemic control is closely linked to poor periodontal health, and that periodontitis contributes to increased insulin resistance in diabetic patients. Furthermore, periodontal disease is associated with a higher risk of diabetes complications, including mortality. Importantly, periodontal therapy has been shown to improve serum HbA1C levels without posing safety concerns. However, this report mainly focused on type 2 diabetes and did not address the specific association between periodontitis and glycemic control in type 1 diabetes, leaving a gap in the evidence regarding the impact of periodontal disease on blood sugar regulation in type 1 diabetics. Type 1 diabetes, being an autoimmune condition with distinct pathophysiology compared to type 2 diabetes, may present different interactions with oral health. Type 1 diabetics often have a younger onset and better overall glycemic control due to insulin therapy, yet they may still experience significant periodontal complications.

In lack of evidence to answer the question: In type 1 diabetes, is periodontitis associated with poor glycemic control? Many studies have focused on the dysfunction of polymorphonuclear neutrophils (PMNs) in individuals with diabetes, particularly in relation to the prevalence and severity of periodontal diseases (23). The dysfunction of these immune cells contributes to a compromised immune response, which is a significant factor in the increased susceptibility to oral infections in diabetic patients. The dysfunction of PMNs in individuals with Type 2 diabetes has been a focal point in understanding the relationship between diabetes and periodontal diseases. These immune cells are significantly impaired in diabetic patients. This dysfunction leads to a compromised immune response, which, in turn, increases the risk of oral infections such as gingivitis and periodontitis (23).

In healthy individuals, PMNs are essential for controlling microbial growth and facilitating the resolution of infections. However, in diabetic individuals, PMNs’ ability to migrate to infection sites, phagocytose pathogens and clear the infection is impaired, allowing pathogens to persist and further aggravate periodontal inflammation (23).

Periodontal inflammation, as seen in conditions like gingivitis and periodontitis, leads to the production of a variety of circulating pro-inflammatory cytokines. These cytokines, including TNF-α, IL-1β, and IL-6, not only exacerbate periodontal tissue destruction but also promote insulin resistance. This creates a dangerous cycle in which poor glycemic control fuels periodontal disease and the resulting oral inflammation worsens the body's ability to control blood sugar levels, making the management of diabetes even more difficult. As a result, periodontal disease becomes not only an oral health issue but also a key factor in the systemic complications of diabetes (24, 25).

Several studies have indicated that type 2 diabetes is a significant risk factor for gingivitis and periodontitis, with the severity of these conditions being closely tied to poor glycemic control (25).

It has been reported that the risk of developing periodontitis in diabetic patients is three times higher than in the general population, further underscoring the importance of managing both oral health and diabetes effectively.

The mechanisms linking type 2 diabetes and oral pathologies, particularly periodontal diseases, are well-documented and share similarities with those involved in other common complications of diabetes, such as retinopathy and nephropathy:

- Hyperglycemia and oral environment (26): Chronic hyperglycemia plays a crucial role in the development of oral diseases. It leads to a decrease in the pH level of the oral cavity, creating an acidic environment that facilitates the growth of harmful bacteria. Additionally, hyperglycemia increases glucose levels in both saliva and gingival fluid, which provides an abundant nutrient source for oral bacteria. These conditions create an ideal environment for the growth of pathogenic bacteria that contribute to gingivitis and periodontitis.

In addition to creating an acidic environment, chronic hyperglycemia also impairs the body's natural defense mechanisms in the oral cavity.

- Formation of Advanced Glycation End-products (AGEs) (27): Another important consequence of hyperglycemia is the formation of AGEs. These are harmful molecules that form when excess glucose reacts with proteins and lipids. The accumulation of AGEs in tissues leads to various detrimental effects, one of the most significant being the promotion of inflammatory processes. The AGEs bind to specific receptors called RAGE (Receptor for Advanced Glycation End-products), which are present on the surface of various cells, including smooth muscle cells, endothelial cells, and monocytes/macrophages. This binding triggers a cascade of inflammatory responses, such as increased production of pro-inflammatory cytokines, which amplify periodontal inflammation and contribute to the worsening of blood sugar control.
- Impact of AGEs on immune and vascular function (27, 28): The interaction between AGEs and RAGEs also affects several key immune and vascular functions. For example the binding of AGEs to RAGEs can increase vascular permeability, promoting the infiltration of immune cells and inflammatory mediators into the periodontal tissues, worsening gum tissue inflammation and destruction.

These mechanisms illustrate the complex interaction between hyperglycemia, AGEs, and the immune response, which together contribute to the heightened risk of periodontal disease in individuals with Type 2 diabetes. The mechanisms linking type 2 diabetes and oral pathologies, particularly periodontal diseases, are well-documented and share similarities with those involved in other common complications of diabetes, such as retinopathy and nephropathy (26, 27, 28).

Given that these same factors are involved in other diabetic complications, managing blood sugar levels and controlling periodontal disease are essential components of improving overall health outcomes for diabetic patients. However, A key limitation of this study is its cross-sectional design, which limits the ability to draw causal inferences. Therefore, the observed associations between Type 1 diabetes and oral health status should be interpreted cautiously. Further studies are needed to clarify the temporal relationships and potential causal mechanisms involved.

## 5 Conclusions

When compared to non-diabetics, our survey unequivocally showed how type 1 diabetes affects children's and adolescents’ dental health.

All participants had at least one dental cavity but the average DMFT/dmft index was higher in diabetic subjects (6.13 ± 3.26). Patients with type 1 diabetes showed a decline in the majority of the oral health metrics examined. The average plaque index for diabetic subjects was (0.97 ± 0.28) and 84% of both groups exhibited gingival inflammation. Tartar deposits were absent in 60% of diabetic subjects and 82% of non-diabetics. Statistically significant differences were identified between diabetic and non-diabetic groups in terms of calculus deposition and degree of inflammation with respectively *p* = 0.001 and *p* = 0.022.

When it comes to assessing the effect of glycemic control on oral health, a correlation was observed between HbA1c and calculus deposit, as well as between HbA1c and the severity of inflammation respectively *p* < 0.001, *p* = 0.01. A statistically significant difference was observed for gingival inflammation which was more pronounced in children with uncontrolled type 1 diabetes (*p* = 0,043). In linear regression a statistically signification relation between the plaque index and tooth brushing in univariate analysis *p* < 0.001.

The results of our study indicated that patients with type 1 diabetes experienced a decline in most of the oral health metrics assessed. This may suggest that both diabetes and its control have a significant impact on oral health

## Data Availability

The original contributions presented in the study are included in the article/Supplementary Material, further inquiries can be directed to the corresponding author.

## References

[1] Bielka W Przezak A Molęda P Pius-Sadowska E Machaliński B. Double diabetes—when type 1 diabetes meets type 2 diabetes: definition, pathogenesis and recognition. Cardiovasc Diabetol. (2024) 23(1):62. 10.1186/s12933-024-02145-x 38341550 PMC10859035

[2] World Health Organization. Global Report on Diabetes. Geneva: World Health Organization (2021). Available online at: https://iris.who.int/bitstream/handle/10665/204871/9789241565257_eng.pdf

[3] American Diabetes Association. Standards of medical care in diabetes—2023. Diabetes Care. (2023) 46(Supplement 1):S1–2. 10.2337/dc23-Sint 36507647 PMC9810461

[4] Preshaw PM Alba AL. Periodontitis in diabetes mellitus: a review of the evidence. Diabetes Metab Res Rev. (2014) 30(2):238–46. 10.1007/s00125-011-2342-y

[5] Chetoui A Kaoutar K Elmoussaoui S Boutahar K El Kardoudi A Chigr F Prevalence and determinants of poor glycaemic control: a cross-sectional study among Moroccan type 2 diabetes patients. Int Health. (2022) 14(4):390–7. 10.1093/inthealth/ihz107 31957782 PMC9248056

[6] Silness J Loe H. Periodontal disease in pregnancy. II. correlation between oral hygiene and periodontal condtion. Acta Odontol Scand. (1964) 22:121–35. 10.3109/00016356408993968 14158464

[7] Weerheijm KL Jälevik B Alaluusua S. Molar-incisor hypomineralisation. Caries Res. (2001) 35(5):390–1. 10.1159/000047479 11641576

[8] Ambildhok K Shetty V. The educational experience of direct observation of procedural skills (DOPS) and traditional assessment methods among dental students and examiners: a comparative study. IJDSIR. (2020) 3(3):479–84. https://www.ijdsir.com/asset/images/uploads/15971594526808.pdf

[9] Singh A Bagadia M Sandhu KS. Spatially coordinated replication and minimization of expression noise constrain three-dimensional organization of yeast genome. DNA Res. (2016) 23(2):155–69. 10.1093/dnares/dsw005 26932984 PMC4833423

[10] Babu GR Murthy GVS Ana Y Patel P Deepa R Neelon SEB Association of obesity with hypertension and type 2 diabetes mellitus in India: a meta-analysis of observational studies. World J Diabetes. (2018) 9(1):40–52. 10.4239/wjd.v9.i1.40 29359028 PMC5763039

[11] Coelho AS Amaro IF Caramelo F Paula A Marto CM Ferreira MM Dental caries, diabetes mellitus, metabolic control and diabetes duration: a systematic review and meta-analysis. J Esthet Restor Dent. (2020) 32(3):291–309. 10.1111/jerd.12562 31912978

[12] Sadeghi R Taleghani F Mohammadi S Zohri Z. The effect of diabetes Mellitus type I on periodontal and dental status. J Clin Diagn Res. (2017) 11(7):ZC14–7. 10.7860/JCDR/2017/25742.10153 28893034 PMC5583944

[13] Surlari Z Ciurcanu OE Budala DG Butnaru O Luchian I. An update on the interdisciplinary dental care approach for geriatric diabetic patients. Geriatrics (Basel). (2023) 8(6):114. 10.3390/geriatrics8060114 38132485 PMC10743251

[14] Ribeiro TR Silva SM Martins RARC Santos CF Silva PGB Forti ACE Salivary immunoglobulin levels and periodontal indices in Brazilian children with and without type 1 diabetes. Braz Oral Res. (2024) 38:e043. 10.1590/1807-3107bor-2024.vol38.0043 38747830 PMC11376677

[15] Shima A Noguchi-Shinohara M Shibata S Usui Y Tatewaki Y Thyreau B Japan prospective studies collaboration for aging and dementia (JPSC-AD) study group. Glucose metabolism and smaller hippocampal volume in elderly people with normal cognitive function. NPJ Aging. (2024) 10(1):39. 10.1038/s41514-024-00164-2 39251602 PMC11384785

[16] Mandura RA El Meligy OA Attar MH Alamoudi RA Dafar AO Rajeh MT Assessment of oral hygiene, gingival, and periodontal health, and teeth eruption among type 1 diabetic Saudi children. Int J Clin Pediatr Dent. (2022) 15(6):711–6. 10.5005/jp-journals-10005-2462 36866125 PMC9973077

[17] Guinan J Meless G Sangaré A Sébastien D Samba M Da-Danho V Analysis of the relationship between oral diseases and glycemic control of diabetes in the west African context: survey at the centre anti-diabétique d’Abidjan (CADA), côte d’Ivoire. Open J Epidemiol. (2018) 8:213–25. 10.4236/ojepi.2018.84017

[18] Vincelet C Levy SA Gremy I. Etat bucco-dentaire et Recours aux Soins Préventifs et Curatifs de la Population Francilienne Adulte. Paris: Observatoire régional de santé d'Ile-de-France (2008).

[19] Parveen S Alhazmi YA. Impact of intermittent fasting on metabolic syndrome and periodontal disease-A suggested preventive strategy to reduce the public health burden. Int J Environ Res Public Health. (2022) 19(21):14536. 10.3390/ijerph192114536 36361416 PMC9657467

[20] Mirnic J Djuric M Brkic S Gusic I Stojilkovic M Tadic A Pathogenic mechanisms that may link periodontal disease and type 2 diabetes Mellitus-the role of oxidative stress. Int J Mol Sci. (2024) 25(18):9806. 10.3390/ijms25189806 39337292 PMC11432179

[21] Graziani F Gennai S Solini A Petrini M. A systematic review and meta-analysis of epidemiologic observational evidence on the effect of periodontitis on diabetes. J Clin Periodontol. (2018) 45:167–87. 10.1111/jcpe.12837 29277926

[22] Chapple IL Genco R. Working group 2 of the joint EFP/AAP workshop. Diabetes and periodontal diseases: consensus report of the joint EFP/AAP workshop on periodontitis and systemic diseases. J Periodontol. (2013) 84(4 Suppl):S106–12. 10.1902/jop.2013.1340011 23631572

[23] Vidya K Shetty P Anandakrishna L. Oral health and glycosylated hemoglobin among type 1 diabetes children in south India. J Indian Soc Pedod Prev Den. (2018) 36(1):38. 10.4103/JISPPD.JISPPD_330_16 29607837

[24] El-Makaky Y Shalaby HK. The effects of non-surgical periodontal therapy on glycemic control in diabetic patients: a randomized controlled trial. Oral Dis. (2020) 26(4):822–9. 10.1111/odi.13256 31834660

[25] Reis-Prado AHD Paula KDS Nunes GP Abreu LG Cintra LTA Peixoto IFDC Top 100 most-cited papers on diabetes mellitus in dentistry: a bibliometric study. Braz Oral Res. (2024) 38:e075. 10.1590/1807-3107bor-2024.vol38.0075 39109771 PMC11376656

[26] Zhang W Zhang S Dong C Guo S Jia W Jiang Y A bibliometric analysis of RNA methylation in diabetes mellitus and its complications from 2002 to 2022. Front Endocrinol (Lausanne). (2022) 13:997034. 10.3389/fendo.2022.997034 36157472 PMC9492860

[27] Melo G Flausino CS Darella IK Miguel AF Martins PA Júnior, Rivero ER. Top 100 most-cited articles on intraoral squamous cell carcinoma and its risk factors: a bibliometric study. Braz Oral Res. (2022) 36:e030. 10.1590/1807-3107bor-2022.vol36.0030 35170696

[28] Baldiotti AL Amaral-Freitas G Barcelos JF Freire-Maia J Perazzo MF Freire-Maia FB The top 100 most-cited papers in cariology: a bibliometric analysis. Caries Res. (2021) 55(1):32–40. 10.1159/000509862 33341798

