# Supplementary material for: Assessment of oral health status in a population of Moroccan children with type 1 diabetes
Source: Front Oral Health. 2025 Sep 12;6:1638222. doi: 10.3389/froh.2025.1638222 (PMC12463878; doi:10.3389/froh.2025.1638222)
Supplement: Supplementary file 2 [file Supplementaryfile2.pdf]

Appendix 2: Non-Diabetic form

Rabat the: ……………………………… form N: ……………………………………..

1. Civil status:

- First and last name:
- Age:
- Gender: male ☐ female ☐

- Educational level of the child: preschool ☐ elementary school ☐ secondary ☐

2. Oral health:

1.dental cavity 2. Missed teeth 3. Filled teeth. 4. White spot lesion. 5.MIH

18	17	16	15/ 55	14/54	13/53	12/52	11/51	21/61	22/62	23/63	24/64	25/ 65	26	27	28
48	47	46	45/ 85	44/84	43/83	42/82	41/81	31/71	32/72	33/73	34/74	35/ 75	36	37	38

MIH yes ☐ no ☐

Periodontal condition:

- The Silness and Löe plaque index (PI)

	D	M	V	L	Score
55/16					
52/12					
64/24					
75/36					
72/32					
84/44					

- The Löe and Silness gingival index (GI):

	D	M	V	L	Score
55/16					
52/12					
64/24					
75/36					
72/32					
84/44					

Presence of calculus: no calculus ☐ moderate ☐ very abundant ☐

its localization: supragingival ☐ subgingival ☐

Index:

**DMFT/ dmft**	**PI (T/24)**	**GI (T/24)**
